# *In Vitro* assessments of ENDS toxicity in the respiratory tract: Are we there yet?

**DOI:** 10.1016/j.namjnl.2025.100016

**Published:** 2025-04-03

**Authors:** Thivanka Muthumalage, Emma Sarles, Qixin Wang, Edward Hensel, Thomas Hill, Irfan Rahman, Risa Robinson, Andrea M. Stroup, Krista Thongphanh, Lisa A. Miller

**Affiliations:** a School of Health Sciences, Purdue University, West Lafayette, IN 47907, USA; b Department of Mechanical Engineering, Rochester Institute of Technology, Rochester, NY 14623, USA; c Department of Environmental Medicine, University of Rochester Medical Center, Rochester, NY 14642, USA; d Office of Science, Center for Tobacco Products, US Food and Drug Administration, Silver Spring, MD, 20993, USA; e Behavioral Health and Health Policy Practice, Westat, Rockville, MD, 20850, USA; f California National Primate Research Center, Department of Anatomy, Physiology, and Cell Biology, School of Veterinary Medicine, University of California, Davis 95616, USA

**Keywords:** EndNDS, E-cigarette, Vaping, Lung, Epithelium, Culture

## Abstract

Approximately 4.6 % of U.S. adults over the age of 18 use e-cigarettes, which are a type of electronic nicotine delivery system (ENDS). Over 2.5 million U.S. middle and high school students also use both disposable and/or flavored ENDS products. The health impacts of ENDS use by adults and adolescents are considered a controversial topic in the social media partially due to misperceptions surrounding ENDS toxicity compared to that of combustible cigarettes. There is growing evidence that ENDS, particularly their product composition and design, individual and combined ingredients, and produced aerosols, are toxic to human health. Animal studies have been critical for defining the pathophysiologic outcomes resulting from ENDS use. However, *in vitro* approaches using human cells can measure the potential toxicity of ENDS e-liquids and aerosols on a shorter timeline and are in keeping with recent statements to replace, reduce and refine the use of animals in biomedical research and regulatory decision making. This review examines current research related to cell culture models of the respiratory tract and exposure methodologies for ENDS use and compares known *in vivo* parameters of injury and inflammation associated with ENDS to different *in vitro* systems developed to replicate the inhaled toxicant outcomes. The design and interpretation of exposure methodologies and technological gaps in the evaluation of ENDS aerosols are also discussed. Given the ongoing evolution and popularity of ENDS products, *in vitro* assessments for measuring respiratory tract injury and inflammation resulting from ENDS use provide a critical scientific platform for rapid evaluation of potential inhalation toxicity in tobacco regulatory science.

## Introduction

Electronic nicotine delivery systems (ENDS) were invented decades ago and are considered an alternative nicotine delivery method to smoking combustible tobacco. Although some have argued that ENDS use is more effective than smoking cessation-counseling or nicotine replacement therapy for tobacco abstinence, the U.S. Food and Drug Administration (FDA) has not approved an ENDS for therapeutic use as a cessation product (Auer et al. 2024a; Auer et al. 2024b; Hajek et al. 2019). In contrast to the traditional combusted tobacco product, ENDS convert a nicotine containing solution (e-liquid) to an aerosol using a heated metal coil that can then be inhaled and exhaled to mimic cigarette smoking. In 2020, there were multiple different ENDS devices available for purchase on the U.S. market, and over 14,000 e-liquid products under 11 main flavor categories (Ma et al. 2022).

The flavoring chemicals added to e-liquids were intended to reduce the sensation of smoke inhalation referred to as “throat hit,” but unexpectedly encouraged initiation of adolescent use with characterizing flavors not traditionally found in combustible cigarettes (Odani et al. 2020). When surveyed in 2020, approximately 83 % of U.S. adolescents who use ENDS preferred fruit-flavors over other flavorings in e-cigarette products (Wang et al. 2020d). Adults also prefer flavored e-cigarette products, with fruit and dessert predominating over tobacco and menthol flavors (Farsalinos et al. 2023). On February 6, 2020, the FDA announced an enforcement prioritization policy on the manufacture, distribution, and sale of unauthorized, flavored cartridge-based ENDS, except for menthol and tobacco flavored products (FDA 2020). However, many flavored ENDS remain available for purchase both in-stores and online. The 2022 National Youth Tobacco Survey (NYTS) estimated that over 2.5 million middle and high school students used ENDS, while findings from the 2024 NYTS estimate 1.63 million students are current users (Jamal et al. 2024; Park-Lee et al. 2022). Although these data suggest a recent decline in use of ENDS products by youth, 4.6 % of adults over 18 also use ENDS, suggesting continuing appeal of and access to ENDS products (Kramarow and Elgaddal 2023). As such, investigating the complex toxicology profile of ENDS aerosols, particularly from newer devices and e-liquid combinations, is desirable to identify hazards and support continued science-based public health decisions.

Some observational studies in human populations have suggested that ENDS use might be less harmful than cigarette smoking (Huerta et al. 2017), and commercial marketing for ENDS has attempted to minimize the perception of hazards associated with these products. However, numerous *in vivo* animal and *in vitro* cell culture studies have reported an adverse physiologic impact of ENDS aerosol exposures (reviewed in (Masso-Silva et al. 2021)). Either acute or chronic exposure to ENDS aerosol increases local inflammatory cytokine levels and recruits leukocytes into the airways (Bahmed et al. 2019; Wang et al. 2019; Wang et al. 2020c). Animal studies have also shown that exposure to ENDS aerosol during pregnancy might impair the *in-utero* development of the fetal respiratory system and induce proinflammatory responses in any surviving offspring (Chen et al. 2018; Wang et al. 2020b). The results from animal studies provide strong experimental evidence for the toxicologic impact of ENDS use on the respiratory tract, but study duration, as well as the sheer number of e-liquid formulations and different commercial ENDS devices make e-liquid/device pairings in animal models impractical for conducting comprehensive assessment of toxicity profiles across ENDS products. Novel *in vitro* models using human cells can be used to investigate the toxicity of ENDS products with much shorter timelines in a human relevant manner, that largely avoids ethical conflicts regarding animal use (Levy 2012).

*In vitro* and *ex vivo* models for the study of ENDS aerosol toxicity incorporate multiple exposure strategies including (1) direct addition of the e-liquid to cultures; (2) percolating the aerosol through growth media for use on cultures; and (3) direct aerosol exposure of cultures, either to cells immersed in media or on air-liquid interface cell culture inserts. Different cell types (*e.g*., epithelium, endothelium, leukocytes, and fibroblast), individually or in combination, have been studied in culture, and increased inflammatory responses have been identified in multiple cell types using these exposure methods (Masso-Silva et al. 2021). There are limitations to the interpretation of findings between some studies using cells derived from the respiratory tract, due to differences in exposure methods, exposure duration, and puff topography used to generate the ENDS aerosol. Traditional two-dimensional (2D) cell cultures do not absolutely replicate the microenvironment of the intact lung *in situ*, as the airways consist of epithelium, fibroblasts and vascular cells interacting within an extracellular matrix. Recent advances in three-dimensional (3D) cell cultures offer an improved ability to restore this morphological and physiological microenvironment of the respiratory tract *in vitro*, which is supported by study findings comparing gene and protein profiles of lung tissue, 2D, and 3D cell culture systems (Fang and Eglen 2017).

The purpose of this review is to examine the most current cell culture systems that are used to conduct toxicology studies of the respiratory tract and compare ENDS exposure systems (both commercial and non-commercial) developed for *in vitro* studies. The state of the science for biomarkers of *in vivo* lung injury and inflammation associated with inhalation exposures are discussed to establish their validation as relevant outcome measures for biomarkers of respiratory toxicity associated with ENDS use. Lastly, future directions and challenges for developing additional *in vitro* models of the respiratory tract for the purpose of conducting tobacco regulatory science are explored.

## *In vivo* biomarkers of ENDS-Mediated lung injury and inflammation

The harm-reduction designation for ENDS is based on the absence of tobacco leaf combustion, which eliminates the presence of tar and particulates generated in the aerosol of traditional cigarettes (Fernandez et al. 2015). In contrast, ENDS generate a heated, volatilized aerosol from an e-liquid solution of carrier solvents (propylene glycol and glycerin), nicotine (synthetic or plant-extracted), combinations of artificial chemical flavorants, and other additives (Dinakar and O’Connor 2016). *In vitro* and *in vivo* animal studies support an association between biomarkers of exposure to ENDS and lung disease, however clinical data obtained from humans predominantly reflects acute respiratory conditions; this is likely due to the comparatively short (<20 years) market availability of ENDS relative to the over 100 years of archival clinical data from subjects who used combustible tobacco cigarettes (Giovacchini et al. 2022). Mechanical exposure systems for experimental testing of ENDS by researchers can simulate human puffing profiles, but factors such as breathing patterns (nose breathing *vs*. mouth breathing, exhalation, and retention), device voltage, single-coil *versus* double-coil, and coil heating preferences are known to affect toxicologic risks of ENDS and increase detrimental impacts on human health (Beauval et al. 2019; Hua et al. 2023; Robinson et al. 2020; Sleiman et al. 2016; Zhou et al. 2021).

Most of the chemical mixtures used as flavorants in e-liquids have their origins in processed commercial food manufacturing. However, their use in food has no bearing on the toxicological evaluation of additives in tobacco products due to the route of exposure (ingestion *versus* inhalation). Further, the toxicity of these compounds is significantly altered when heated, volatilized, and partially combusted prior to inhalation into the respiratory tract. For example, occupational exposure of food workers to the chemical diacetyl, which has been used as a butter flavorant in commercial popcorn, is known to cause severe lung diseases such as bronchiolitis obliterans when volatilized and inhaled (Kanwal et al. 2006). The use of heat to convert chemical flavorings into an aerosol can also generate reactive carbonyls which are known to damage lung tissue upon inhalation (El-Hellani et al. 2018; Ogunwale et al. 2017). Chemical analyses of ENDS aerosols generated by many sweet-flavored e-liquids has confirmed the production of high levels of aldehydes (Gillman et al. 2020). Acute exposures of mice to ENDS aerosols from flavored e-liquids elicits significant oxidative stress in the lung and other organs compared to exposures of propylene glycol/vegetable glycerin solvents alone (Been et al. 2022; Lamb et al. 2022; Moshensky et al. 2022; Wong et al. 2022). Despite federal and state restrictions on sales of flavored e-liquids, these products remain commercially available for purchase on the internet. The custom blending of multiple-liquid flavors by the user for use in “open tank” ENDS is a common activity in both adults and adolescents, however there are currently little experimental data evaluating if these aerosols increase the risk of adverse respiratory symptoms and lung disease (Morean et al. 2018).

Elevated levels of glutathione, a cellular tripeptide antioxidant, is significantly decreased in lung homogenates of rats that were acutely exposed to unflavored ENDS aerosol (Wang et al. 2020a). Along with this evidence of oxidative stress, several potential biomarkers predictive for membrane damage and cell death have been identified in rodent models of ENDS aerosol exposures, including lactate dehydrogenase (LDH) and alkaline phosphatase (ALP) enzyme in bronchoalveolar lavage fluid (BALF) (Day et al. 2023; Muthumalage and Rahman 2023). Altered expression of inflammatory mediators in BALF, such as KC, IL-6, and TNFα, have been reported in acute ENDS aerosol exposures, indicating the local induction of immune cell activation (Moshensky et al. 2022; Muthumalage and Rahman 2023). A study by Szafran, et al., showed increases in CD+4 T cells and CD+19 B cells in BALF of mice post exposure to unflavored or vanillin-flavored ENDS aerosol, in addition to increases in 12-hydroxyeicosotetraenoic acid (12-HETE) and 2-arachidonylglycerol (2-AG), which suggests that e-liquid formulations impact immunoregulatory pathways in the lung (Szafran et al. 2020). Additional immune-related biomarkers of acute lung injury, such as reduced surfactant proteins, TRPA1 receptor signaling, alterations in NLR3 inflammasome, and PAI1 protein levels have been demonstrated in lung homogenates from two different mouse strains at 24 h post-ENDS aerosol exposure (Muthumalage and Rahman 2023).

Chronic exposure (5 days/week) of mice to mint or mango-flavored ENDS aerosols for 6 months resulted in an increase in circulating proinflammatory cytokines but no significant differences in lung inflammation or visible histopathology were reported (Crotty Alexander et al. 2018). Despite the lack of substantial cellular pathology from chronic ENDS aerosol inhalation, molecular differences in proinflammatory cytokines and immune parameters are detectable in BALF and whole lung homogenates (Crotty Alexander et al. 2018; Masso-Silva et al. 2021). In contrast, others have reported substantial lung inflammation and histopathology in mice after 9 weeks of ENDS aerosol exposure to a peach flavored e-liquid, which would be consistent with the finding of increased BALF neutrophil elastase and metalloproteinase activity in humans who use ENDS or tobacco cigarettes (Esquer et al. 2022; Ghosh et al. 2019). Mouse exposure studies have also reported decreased pulse oximetry and lung function upon long-term exposure to ENDS, in a manner similar to that seen in human subjects (Larcombe et al. 2017; Wei et al. 2021). Finally, exposures to both nicotine-containing ENDS aerosol or tobacco smoke have been reported to upregulate the expression of nicotine acetylcholine receptors (nAChRs) in airway epithelium, as well as increase mucin production and mucus histochemical staining in mouse lungs (Gundavarapu et al. 2012).

Despite enforcement of state and federal regulations on the marketing and sale of ENDS, unsanctioned user modification of devices and spiking of e-liquids with other substances has introduced new exposure hazards and associated biomarkers of effect. Electrical alterations that increase the coil temperature used to volatilize e-liquids is used to enhance the aerosol “hit”, and the spiking of e-liquids with the addition of cannabidiol (CBD), tetrahydrocannabinol (THC) or other illicit substances has further complicated the ability of health care providers and public health officials to assess health effects of these exposures. Urinary and plasma biomarkers, such as nicotine and THC metabolites, have now been leveraged to assess absorption and distribution of e-liquid ingredients in human subjects (Boykan et al. 2019; Farsalinos et al. 2014; Podguski et al. 2022). In non-clinical studies, CBD oils were detectable in mouse BALF, blood and urine, indicating studies of nicotine and THC/CBD effects in animal models might be used to generate dose-response estimates between exposure levels and health outcomes (Bhat et al. 2023). Putative carcinogens such as formaldehyde, acetaldehyde, acrolein, and silicate, have also been detected in the saliva and urine of people who use ENDS, which suggests that prolonged inhalation of heated and aerosolized e-liquids could lead to more severe health outcomes in the future (Keith et al. 2020; Marques et al. 2021; Mizumoto et al. 2017; Poinen-Rughooputh et al. 2016; Protano et al. 2021; Shahab et al. 2017).

## Respiratory tract cell cultures

Studies using *in vitro* models can expedite toxicity profiling for ENDS products (including e-liquids and devices) by shortening experimental timelines and lowering the high capital cost incurred when using *in vivo* models, as well as enhance the mechanistic understanding of cell-specific effects (Langhans 2018). In 2014, the first report of *in vitro* studies to model the effects of ENDS use in the lung parenchyma employed immersed cultures of primary human tracheobronchial epithelial cells and direct application of ENDS e-liquid into the medium as a controlled exposure strategy (Wu et al. 2014). Subsequent studies have used similar direct exposures with a range of cell culture platforms, including epithelial lung cell lines to assess ENDS product toxicity (Rowell et al. 2017; Sherwood and Boitano 2016). As discussed later in this review, *in vitro* exposure systems have been designed and characterized to permit evaluation of aerosolized ENDS e-liquids, including those produced by fourth generation ENDS devices.

When investigating for cellular and molecular toxicity resulting from ENDS aerosol inhalation, the primary cell types of interest are the epithelial cells lining the respiratory tract, which serve as an essential barrier to protect the lung from injury due to pathogens or toxicants. Traditional 2D monolayer cultures used in the assessment of inhaled exposures may consist of either immortalized lung cell lines (A549, BEAS-2B, Calu-3, NCI-H292) or primary lung epithelial cell isolates prepared from donor post-mortem tissues or living patient biopsies (Yaqub et al. 2022). A distinguishing feature of the 2D cell culture platform is the growth of cells (fully or partially immersed) in a growth medium on a rigid polystyrene surface. Further refinement of this approach incorporated a porous cell culture insert to permit diffusion of media components to the basolateral perspective of primary tracheobronchial isolates on an air-liquid interface (ALI) insert, resulting in a more differentiated epithelial population that included ciliated and goblet cells (Gray et al. 1996). However, this arrangement does not account for the complex tissue architecture or comprehensively capture the intercellular interactions between the mucosal wall tissues of the airway. Because of these biomechanical limitations, 2D cultures have a limited ability to recapitulate *in vivo* outcomes, but the relative simplicity and consistent reproducibility of such systems makes them attractive for use in preliminary screening assays for ENDS e-liquids or aerosol exposures.

To address the tissue architecture issues of 2D cell culture platforms, complex 3D cell culture approaches have been developed for the conducting airways and gas-exchange regions of the lung. The initial advancement of the field of 3D cell cultures consisted of growing conducting airway epithelium on collagen gels, to structurally replicate the lumen of the mammalian lung and its air-liquid interface (de Jong et al. 1994). Advances in available 3D culture systems now include lung epithelial cells layered on fibroblasts and pulmonary endothelia using proprietary methodology to recapitulate structure of the airway mucosa (Barosova et al. 2020). Other approaches have leveraged specialized gel matrices with highly characterized media supplements that support spheroid and organoid cultures of human alveolar epithelium with the capacity to differentiate into both type I and type II cells (Katsura et al. 2020).

In addition to improvements in maintaining *in vivo*-like differentiated phenotypes in the culture of primary cells, there are innovative approaches that build upon the 3D strategy for lung cell growth. Organ-on-chip platforms integrate microfluidics with cell cultures to capture phenomena such as shear stress and mechanical stretching that can occur in the lung parenchyma after chemical or environmental challenges (Ma et al. 2021). The influence of the physical mechanics of respiration on epithelial cell function using lung-on-a-chip were first investigated by Huh et al., who used combined cultures of epithelial and endothelial cells in the chip platform (Huh et al. 2010). Since then, the use of lung-on-a-chip has been successfully applied to studies of toxicant exposure and disease models, including COPD and SARS-CoV-2 infection (Benam et al. 2016; Tang et al. 2020). Along with lung-on-a-chip, human inducible pluripotent stem cells (iPSC) have increasingly been incorporated into lung disease models. Human iPSCs can differentiate into multiple lineages that lead to organ development through manipulation of key signaling pathways in a carefully orchestrated culture system (Blau and Daley 2019). Differentiation of iPSC cultures into primordial lung tissue requires time and mediator-dependent induction of definitive endoderm, followed by anterior foregut endoderm specification, leading to branching morphogenesis and epithelial cell lineage specification (Morrisey and Hogan 2010). Significantly, cell cultures derived from human iPSC lines have been reported to differentiate into either proximal or distal airway epithelial cell lineages, with a transcriptome that appears to be more comparable to primary cell cultures than the transcriptome of immortalized lung cells (Hawkins et al. 2021; Huang et al. 2015; Jacob et al. 2017; Leibel et al. 2019).

Initially, cellular toxicity resulting from ENDS use was studied by adding ENDS e-liquid to mouse stem cell and human fibroblast cultures (Bahl, Lin et al. 2012), until it was shown that e-liquid also introduces harmful thermal degradation byproducts into the aerosol when volatilized by the heated coil of the ENDS device (Lee, Szulejko et al. 2018). Both cellular injury and inflammatory responses after ENDS aerosol exposure have been evaluated in 3D cultures of respiratory tract epithelium; while difficult to directly extrapolate into animal models, the overall profile of is one of moderate cytotoxicity (Aug et al. 2015; Ganguly et al. 2020; Wang et al. 2019; Wick et al. 2022). Of particular significance, an iPSC cell line differentiated towards a type 2 distal airway cell phenotype in a 3D culture that was exposed to ENDS aerosol, induced the proinflammatory cytokine IL-8, cytochrome P450 enzymes and a transcriptomics profile that was similar to the responses of cells exposed to combusted tobacco smoke (Abo et al. 2022). These findings are comparable to earlier studies using primary cultures of human nasal epithelium, which reported similar functional dysregulation of ciliary beat frequency with exposure to either tobacco smoke or ENDS aerosol (Carson et al. 2017). It should be noted that common tobacco humectants such as propylene glycol and glycerol are universally used as carrier solvents in ENDS e-liquids and ENDS-generated aerosols containing these compounds have been reported to induce toxic effects in human lung cell lines and ALI human airway epithelial cell cultures (Escobar et al. 2020).

## *In vitro* ENDS exposure systems for respiratory tract cultures

*In vitro* systems for assessing toxicity of tobacco products in the respiratory tract traditionally focus on either analytic emissions testing or acute biological effects and biomarkers and may be further categorized as commercial or in-house systems. Analytical emissions testing focuses on the capture and analysis of aerosol to identify and quantify the presence of constituents present. Biological testing focuses on exposure of biological samples, such as mammalian cells, bacteria, or animals, to tobacco product emissions and subsequent assays of toxicity, oxidative stress, genotoxicity, and screening biomarkers. Both objectives require emissions systems to actuate the ENDS device or combust the leaf-based product and carry the resultant ENDS aerosol or tobacco smoke to the emissions capture device(s) or biological exposure subsystem.

The first smoking machine appears to have been used in 1892 when the Connecticut Agriculture Experiment Station puffed a cigar using a siphon, and actual smoking machines developed for testing combustible tobacco products were introduced between 1939 and 1971 (Klus et al. 2016). ENDS toxicology testing technology has evolved as a modification of this equipment originally designed for combustible tobacco products. Thorne and Adamson of British American Tobacco recently reviewed three commercially available cigarette smoking machines (Borgwaldt, Burghart, and Vitrocell) and exposure chambers (Curbridge Engineering/ British American Tobacco, Cultex, and Vitrocell) and four exemplar in-house systems (Thorne and Adamson 2013). A common theme of both combusted cigarette and ENDS smoking machines is that they and their exposure chambers are two separate, but connected, devices. Our review of these platforms focuses on the recent innovations in toxicology testing that offer physiologically relevant puffing and mixing profiles, improved dosimetry and exposure conditions that are more reflective of exposure to the tobacco product end user.

Tobacco cigarette smoke exposures using ALI cell culture platforms were developed in an effort to mimic the microenvironment of the airways (Klus et al. 2016). ALI was first integrated with an emissions system in 2002 to study exposure of human bronchial epithelial cells HFBE21 and lung fibroblasts LK004 to three different environmental atmospheres, including side-stream cigarette smoke (Aufderheide et al. 2002). Since then, use of ALI exposure chambers connected to emissions systems have become routine for biological testing of tobacco smoke and ENDS aerosols. The three commercially available ALI exposure systems most commonly reported in the literature are Aufderheide’s CULTEX^®^ system, the Vitrocell^®^ system, and British American Tobacco’s exposure chamber coupled with Borgwaldt emissions systems (Li 2016). The SciReq expoCube^®^ is a more recently developed exposure system that uses thermophoresis to control and increase surface (epithelium) particle deposition (Gindele et al. 2020).

Current emissions systems used for biological (Aufderheide and Mohr 2000; Fields et al. 2017; Neilson et al. 2015) and analytical testing (Hensel et al. 2018) were not designed to produce biomimetic deposition patterns. Recent publications have documented efforts to integrate higher levels of biomimicry into tobacco product testing by integrating the exposure chamber with the emissions platforms; devices have been developed to integrate aerosol generation, distribution, and environmental control. For example, Biomimetic Smoking Robot (BSR) regulates particle dispersion between the ENDS output and the exposure chamber and is the first system reported to deliver volumes small enough for organ chips (Benam et al. 2020). The dose exposed to the organ chip in the BSR is limited to geometries and flow conditions found in the lower lung. The BSR creates puffs at constant flow rate within a trial, permits flow rate manipulation between trials, and has a maximum puff volume of 80 mL. As of this date, the BSR has been validated using an airway chip, but no other exposure detection platforms.

The inHALES system was developed by Philip Morris International R&D and described in a 2020 publication (Steiner et al. 2020). The inHALES is the first emissions system reporting an ability to simulate regular breathing between puffing. The authors incorporated a flow path modeling bronchial branches and diameters representative of six generations of the human airway. Bidirectional flow that models inhalation and exhalation cycles is accomplished using primary and secondary pumps to create variable flow rates during a puffing trial. The system exposes ALI cell culture inserts supported on hydrogel scaffolds at various locations perpendicular to the airflow path to approximate *in vivo* shear stress on cells in conducting airways of the lung. At this time, the inHALES system is a proof-of-concept device described only in Phillip Morris literature, that has been tested only using clean air controls.

The inHALES system has had notable issues and limitations regarding cell culture viability. While biomechanical cues, such as fluid shear, are known to alter protein expression (Orr et al. 2006), only the BSR and inHALES biomimetic systems propose to account for effects of fluid shear on the biological response to aerosol exposures. Steiner et al. cultured cells on ALI inserts until maturity, then punched the tissue culture from the membrane, and further cultivated the tissue culture on a hydrogel matrix (Steiner et al. 2020). The hydrogel-matrix tissue cultures were placed in cavities of the inHALES exposure system, flush with the inner wall and perpendicular to the airflow. While the method shows promise for further development, study exposures to lab air at physiologically appropriate flowrates generated inconsistent results and the culture technique had poor cell survival outcomes.

Organ-on-a-chip technology has demonstrated impressive replication of the microenvironment of small airway tissue (Benam et al. 2020; Huh et al. 2010; Stucki et al. 2015). Benam et al. established shear stress on cells during either cigarette smoke or ENDS aerosol exposures with an airway-on-a-chip platform (Benam et al. 2020). However, the plastic airway-on-a-chip platform is not practical for modeling the entire human airway, as stiffness of the extracellular matrix is a significant contributor to modeling biologically relevant culture conditions. Fibroblasts, epithelium, and endothelial cells in the lung are reported to alter morphologies and phenotypes in response to the stiffness of the extracellular matrix (Zhou et al. 2018). The effect of the matrix on study findings can be dramatic. Using human upper airway epithelial cells cultured on soft scaffold materials, Tapparel et al. found that human rhinoviruses tend to enter and exit only at the apical cell surface (luminal side) of the cultured tissue. These findings would not have been possible without an anatomically relevant 3D culture to replicate the physiologic microenvironment of the softer lung parenchyma (Tapparel et al. 2013).

The field of inhalation toxicology has a shortage of *in vitro* platforms for study of lung injury and disease. Exposure systems which permit multi-scale modeling of human airways while controlling mechanistic cues, such as bidirectional wall shear stress, under repeatable dose conditions are needed to understand the driving mechanisms and adverse outcomes of inhaled toxicants. Systems which can be configured to support sampling for analytical chemistry in tandem with biological monitoring area also need to establish correlations between mass distribution, particle size distribution and species distribution throughout the airway, as a tool to optimize the biologic relevance of inhalation studies and enhance our mechanistic understanding of tobacco and ENDS-related pathophysiology.

## Future directions

Due to the overwhelming number of untested ENDS products already on the market worldwide, there is a need to adopt the use of *in vitro* models to expedite scientific evaluation of these new and evolving tobacco products on respiratory tract physiology and their potential impact as a whole on the public health. Despite impressive improvements for *in vitro* platforms that permit growth and differentiation of primary human lung cells such as ALI and organoid, there are significant harmonization issues with exposure methodology and dosimetry between studies, as well as the use of cell lines outside of the lung-derived epithelial lineage. An additional issue in ENDS respiratory tract toxicity research is an inconsistency in the reporting of outcome measures, making it difficult to compare findings between studies. For example, the toxicological effects of ENDS aerosol exposure has been reported as an IC50 relative to the dosimetry (Singh, Luquet et al. 2016, Behar, Luo et al. 2018, Bishop, Haswell et al. 2019, Wu, Xu et al. 2021), or relative to degree of cell viability (Adamson, Jaunky et al. 2018, Ureña, Ebersol et al. 2020), cytotoxicity (Zanetti, Sewer et al. 2018), or LDH release (Delaval, Egli et al. 2019). Further, the IC50 dose has been reported in mass per unit volume (Bishop, Haswell et al. 2019, Wu, Xu et al. 2021), exposure time (Bishop, Haswell et al. 2019), percent aerosol (Behar, Wang et al. 2018), and percent of puffs (Singh, Luquet et al. 2016). Without agreement on harmonized methodology and outcome reporting, it is difficult to compare ENDS study outcomes and advance the state of the science in respiratory tract toxicology.

The introduction of new, unexamined additives to ENDS e-liquids introduces a need for new analytic chemical methodology to optimize translational validation of biologic dose with absorption and whole animal dosimetry between *in vitro* and *in vivo* models. With the market for non-leaf-based tobacco products continuing to evolve in terms of the number of potential toxicant hazards, more research is needed to determine reference levels and define biomarkers for early detection of lung disease and other long-term health risks. *In vitro* models are moving closer to replication of the mechanical and physiologic conditions of the *in situ* environment within the respiratory tract. Innovation in cell culture techniques specific to exposure of biological samples to inhaled environments can be problematic for grant-funded scientists who rely on established protocols for defensible rigor and may not be free to pursue revolutionary or novel methodologies that enhance translational application of *in vitro* findings. Paradoxically, the potential rigor and defensibility of these new methodologies, not to mention their impact on translational research, might very well equal or exceed that offered by today’s “established protocols.” The truth of this postulate can only be realized through the actuation of novel ideas and their submission as novel methods articles to peer-reviewed journals, and their adoption within the research community. The need for better or novel methodology in respiratory toxicology is not new; it is a situation that is entirely within our collective ability to resolve.

**Fig. 1.**
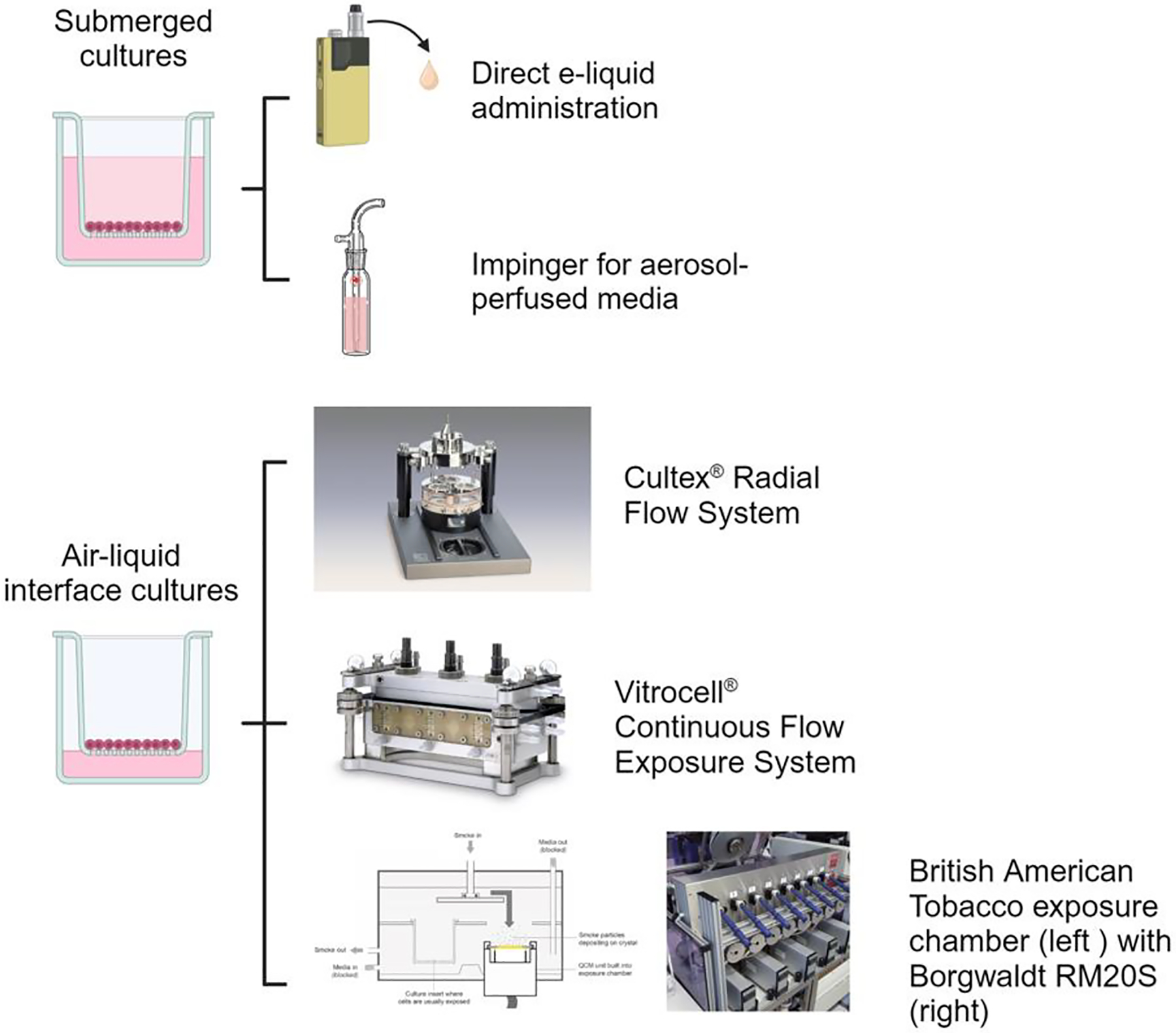
*In Vitro* Models of Respiratory Epithelium and ENDS Exposure Summary.

In the meantime, a problematic challenge for the field is how to integrate existing data collected from the range of *in vitro* culture models and exposure systems (Fig. 1). The sharing of experimental data in the public domain offers the potential for advanced data mining or pattern recognition analysis through public or private means that has been successful with discovery of predictive disease biomarkers for other organ systems (Hill et al. 2017; Hill et al. 2020). General acceptance of translational biomarkers with predictive potential supports enhanced analysis of existing data, as well as drives and optimizes the innovation efforts needed to determine the chemical inhalation hazards in any given tobacco product. The question of whether we are “there” yet remains unanswered, but collaborative efforts to optimize respiratory tract cell culture, improve aerosol exposure systems and harmonize the reporting of toxicity outcomes may ultimately be the most powerful public domain contributions in the short term to lung toxicology and tobacco regulatory science.

## Collaborators

A subgroup of the Toxicity Special Interest Group (SIG) coordinated by the Center for Coordination of Analytics, Science, Enhancement and Logistics (CASEL) in Tobacco Regulatory Science: Lisa Miller, Thivanka Muthumalage, Andrea M. Stroup, Thomas Hill III, Risa J. Robinson, Edward C. Hensel, and Irfan Rahman.

## Data Availability

No data was used for the research described in the article.
